# Investigating the Mechanisms Underlying Cell Death Induction by a Tributyltin Molecule in HTLV-1-Infected Cells Dependent or Not on IL-2 as a Growth Factor

**DOI:** 10.3390/ijms27146165

**Published:** 2026-07-10

**Authors:** Valeria Stefanizzi, Evariste Molimbou, Emanuela Balestrieri, Antonella Minutolo, Franca M. Cordero, Sandro Grelli, Antonio Mastino, Claudia Matteucci, Beatrice Macchi, Francesca Marino-Merlo

**Affiliations:** 1 Department of Chemical, Biological, Pharmaceutical, and Environmental Sciences, University of Messina, 98166 Messina, Italy; valeria.stefanizzi@unime.it (V.S.); fmarino@unime.it (F.M.-M.); 2 Department of Experimental Medicine, University of Rome “Tor Vergata”, 00133 Rome, Italy; emolimbou@gmail.com (E.M.); balestrieri@med.uniroma2.it (E.B.); antonellaminutolo@gmail.com (A.M.); grelli@med.uniroma2.it (S.G.); matteucci@med.uniroma2.it (C.M.); 3 Department of Organic Chemistry “Ugo Schiff”, University of Florence, 50121 Florence, Italy; franca.cordero@unifi.it; 4 The Institute of Translational Pharmacology, Consiglio Nazionale delle Ricerche (C.N.R.), 00133 Rome, Italy; antonio.mastino@ift.cnr.it; 5 Department of Chemical Science and Technology, University of Rome “Tor Vergata”, 00133 Rome, Italy

**Keywords:** HTLV-1, adult T-cell leukemia/lymphoma, organotin compounds, viral oncogenesis, HBZ, cell death

## Abstract

Human T-Lymphotropic virus type 1 (HTLV-1) lifelong infects at least 5–10 million people worldwide, a minority of whom develop severe lethal diseases including adult T-cell Leukemia/lymphoma and HTLV-1-associated myelopathy or tropical spastic paraparesis. Currently, no vaccines or curative therapies to fight HTLV-1 infection or diseases are available. Recently we found that a tributyltin molecule, Bu_3_SnOCOCF_3_ (TBT), which is more potent than cisplatin in inducing cytotoxic effects towards a panel of cell lines including high-tumorigenic cells, also exerted potent cytotoxic effects even towards HTLV-1-infected cell lines, mimicking different states of virus-driven transformation. The type of cell death involved was elusive. In the present study, the effects of TBT on virological and cell death parameters were investigated in HTLV-1-infected immortalized lymphocytes generated by in vitro infection and rendered with or without progressive independence from interleukin-2 as a growth factor. Molecular studies demonstrated that TBT affected HTLV-1 viral gene expression, especially HBZ. TBT confirmed its high cytotoxic potential on the HTLV-1-infected cell lines assayed, especially towards the IL-2-independent HTLV-1-infected cells. Investigation of mechanisms involved in cell death induced by TBT in HTLV-1-infected cells confirmed that caspase 3 and 8 activation, as well as apoptotic response, were relevant. In addition, pyroptosis as well as other unspecifed forms of lytic death presumably contribute to cell death induced by TBT in HTLV-1-infected cells, while a concomitant activation of an autophagic response by this compound seems to mitigate it. Overall, these experimental results outline a particular profile of TBT-induced cell death in HTLV-1-infected cells that is useful for future studies aimed at verifying the real potential of tin-based compounds to contrast diseases caused by HTLV-1.

## 1. Introduction

Human T-lymphotropic virus type 1 (HTLV-1) was the first human retrovirus to be identified in lymphocytes from a patient with cutaneous T-cell lymphoma [1]. Besides having the structural and enzymatic genes common to all retroviruses, HTLV-1 is characterized by a complex expression of regulatory and accessory genes [2]. A recent meta-analysis-based calculation accounts for an estimated number of 82.5 million people infected with HTLV-1 worldwide [3]. Endemic areas of HTLV-1 are generally considered to be Western and Central Africa, Latin America, the Western Pacific Region, and Australia. Mainly due to immigrant flux, however, HTLV-1 has been progressively diffused throughout the world, leading World Health Organization (WHO) to affirm that “more concerted global public health actions are needed to contain this infectious disease” and to organize a meeting on 8–9 December 2025 to provide recommendations and guidance on HTLV-1 testing and prevention [4]. Infection caused by HTLV-1 is mainly transmitted through sexual contact, breast-feeding, and blood-to-blood contact, including via needle sharing and unsafe blood transfusions [5]. Lifelong infection is asymptomatic in most infected individuals, but it can cause an aggressive malignancy of the blood and blood-forming organs, known as adult T-cell leukaemia/lymphoma (ATLL), as well a progressive neurological condition known as HTLV-1-associated myelopathy or tropical spastic paraparesis (HAM/TSP) [5]. CD4+ T cells are the preferential cell targets of HTLV-1 infection. Once it has entered infected cells, the RNA viral genome is retrotranscribed and the DNA provirus is integrated into the host cell DNA, thus establishing the selective maintenance of infected clones in asymptomatic HTLV-1 carriers [6] and lifelong chronic infection, characterized also by infectious spread persistence with alternate blips of cell replication and cell death [7]. There are neither vaccines currently available to prevent HTLV-1 infection nor definitive therapies to cure diseases caused by this virus. Nevertheless, information accumulated in recent years from clinical trials and preclinical studies in vitro and in vivo suggest that combined strategies using various viral and host factors involved in pathogenesis could provide useful tools for fighting HTLV-1 in the future [8,9,10,11,12,13,14,15,16,17,18].

Cisplatin is the most known and widely used metal-containing cancer chemotherapeutic. However, this drug and its derivatives suffer from systemic toxicity and drug resistance concerns. Therefore, in recent years, particular attention has been paid to compounds containing another metal, such as tin, in their structure. These compounds, generally referred to as organotin compounds, have the characteristic of high flexibility in their structures, which enables easy access either to various molecular structures to generate novel derivatives or to cell molecules to produce certain biological effects, including potential anti-tumor activities [19,20,21,22]. Although the mechanisms involved in the anti-tumor activity of organotin compounds have not always been made clear, for some of them, induction of cell death by apoptosis has been hypothesized [23].

Even more scattered are the data regarding the possible direct antiviral effects of organotin polymers against DNA and/or RNA viruses. Regarding the tumor-associated Epstein–Barr DNA virus, results of a study suggest that a triphenyltin compound affected the expression of the BMLF-1 viral gene as a result of its interaction with the Raji cell DNA [24]. On the other hand, organotin polymers derived from diethyltin, dibutyltin, and diphenyltin showed good inhibition of both RNA and DNA viruses [25]. In particular, the possibility of synthesizing organotin polyamine ethers with acyclovir in their backbone that exert antiviral activities towards herpes simplex virus-1 and varicella zoster virus was demonstrated [26].

A first study by some of the current authors demonstrated that tributyltin (IV) exerts cytotoxic activity towards a human head and neck squamous cell carcinoma (HNSCC) cell line even more powerful than that exerted by cisplatin [10]. This compound, however, was not without cytotoxicity towards non-tumor cells and was shown to induce a not-well-characterized form of cell death in which apoptosis and necrosis coexisted. In addition, we have recently shown that an ad-hoc synthesized organotin derivative, tributylstannyl 2,2,2-trifluoroacetate (TBT-OCOCF3, hereafter simply referred to as TBT), is also able to exert a potent cytotoxic effect toward HTLV-1-infected cells at different stages of the virus-driven transformation. Interestingly, the fully transformed C91/PL cell line was quite resistant to TBT-induced cell death, while a continuously growing cell line generated by the authors through HTLV-1 infection and mimicking a pre-malignant IL-2 independent state of infection was more sensitive than uninfected cells to TBT-induced cell death. Nevertheless, information about the type of cell death induced by TBT in HTLV-1-infected cells was elusive [27].

In the present study, we therefore focused our attention on the characteristics of cell death induced by TBT in HTLV-1-infected cells and the possible translation significance of the related findings. Based on previous results, we excluded in this study fully transformed HTLV-1-infected cell lines highly resistant to TBT and concentrated our efforts on HTLV-1-infected cell lines at different pre-malignant stages of virus-induced immortalization/transformation. To this purpose, we utilized an IL-2-dependent cell line, designed as PB2/IL-2, and an IL-2-independent cell line, designed as PB2/NO-IL-2, generated in our laboratory by in vitro infection with HTLV-1 of peripheral blood lymphocytes from a single healthy donor and continuous cell culture in the presence of the IL-2 growth factor or by progressive reduction of IL-2 in the culture medium, respectively, as previously described [27]. These cell lines are equally both CD3 and CD4 positive, but differently express the CD25, CD69, and HLA-DR activation markers, showing PB2/NO-IL-2 cells with higher levels of median fluorescence intensity for all the activation markers than the PB2/IL-2 cells [27]. Thus, PB2/IL-2 and PB2/NO-IL-2 also phenotypically mimic two successive stages of HTLV-1 infection that precede the possible final virus-driven transformation that occur in patients. To define the mechanisms involved in the effects of TBT toward these HTLV-1-infected cells, viral gene expression and various parameters specific to different forms of cell death and their kinetics were investigated.

## 2. Results

### 2.1. Effects of TBT on Viral Gene Expression in HTLV-1-Infected Cells

**Figure 1:**
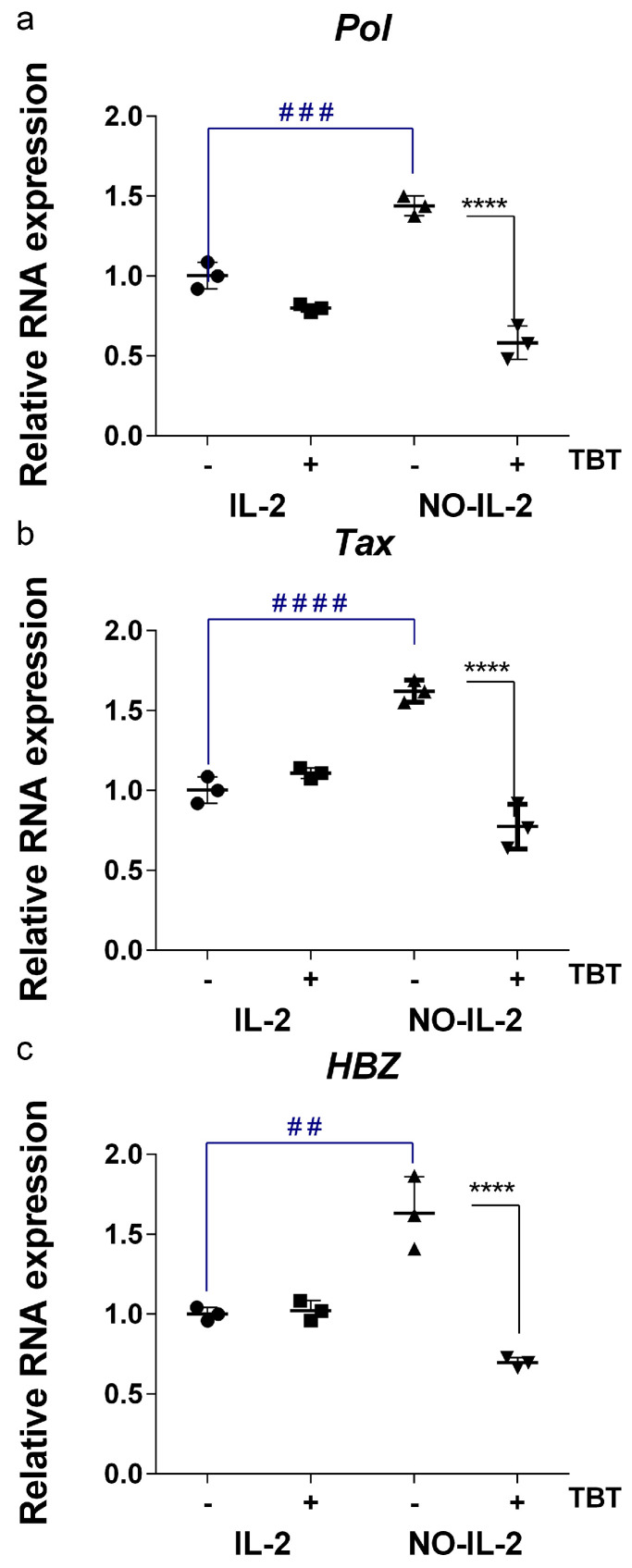
HTLV-1 viral gene expression. Relative RNA levels of Pol (**a**), Tax (**b**), and HBZ (**c**) in PB2/IL-2 and PB2/NO-IL-2 cells treated or not with 5 µM TBT for 6 h, evaluated by RT-qPCR. Expression levels were normalized to 18S rRNA and calculated using the 2^−ΔΔCT^ method. Data represent mean ± SD of three independent experiments. Asterisks indicate comparisons between untreated and TBT-treated samples within the same cell line, whereas hashtags indicate comparisons of basal expression levels between untreated PB2/IL-2 and PB2/NO-IL-2 cells. **** *p* < 0.0001; ## *p* < 0.01; ### *p* < 0.001; #### *p* < 0.0001.

Data published in our previous study showed that TBT-treated HTLV-1-infected cells were prone to die, but an aspect that remained to be clarified was whether a short treatment with this compound could also affect HTLV-1 viral gene expression. To provide information on this issue, 2.5 × 10^5^ HTLV-1-infected cells from the IL-2 dependent PB2/IL2 cell line and from the IL-2 independent PB2/NO-IL-2 cell line were treated with 5 µM for 6 h, and then the expression of the HTLV-1 genes Pol, Tax, and HBZ—as critical virological markers for virus transcription—were analyzed by RT-qPCR. Results are shown in Figure 1 as relative expression to the lowest expressed sample of HTLV-1 genes in PB2/IL-2 and PB2/NO-IL-2 cells. Treatment with 5 µM TBT did not affect the Pol, Tax, and HBZ RNA expression in PB2/IL-2 cells with respect to the untreated counterpart. Conversely, the treatment caused a highly significant (*p* < 0.0001) decrease in Pol, Tax, and HBZ expression with respect to the untreated control in PB2/NO-IL-2 cells. Moreover, a comparison of the basal expression of Pol, Tax, and HBZ in the two cell lines showed significant increases in Pol (*p* < 0.001), Tax (*p* < 0.0001), and HBZ (*p* < 0.01) RNA expression in untreated PB2/NO-IL-2 cells in comparison with corresponding untreated PB2/IL-2 cells. Thus, these results indicate that PB2/NO-IL-2 HTLV-1-infected cells at a more advanced, but tentatively still premalignant, stage of HTLV-1-driven transformation coherently showed more elevated Pol/Tax/HBZ expression compared to PB2/IL-2 cells. Moreover, in addition to a higher sensitivity to cell death proneness induced by TBT, as previously demonstrated [27], PB2/NO-IL-2 cells also showed higher sensitivity to TBT-induced down regulation of viral gene expression than PB2/IL-2 cells.

### 2.2. Effects of TBT on Viability of HTLV-1-Infected PB2/IL-2 and PB2/NO-IL-2 Cells in the Early Phase of Treatment

**Figure 2:**
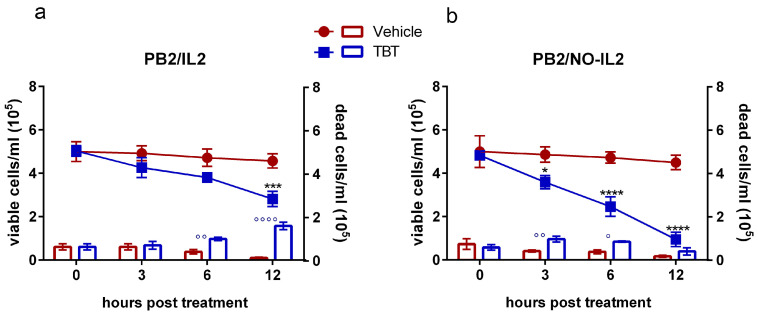
Cell death/viability, detected by the Trypan Blue exclusion test, in PB2/IL-2 (**a**) and PB2/NO-IL-2 (**b**) cell lines, before (0) or after treatment with vehicle or 10 µM TBT for 3, 6, and 12 h. Viable cells (lines, left *y*-axis) and dead cells (bars, right *y*-axis) are reported as mean ± SD from three independent experiments performed in triplicate. Asterisks denote statistically significant differences in viable cell counts and open circles denote statistically significant differences in dead cell counts between TBT-treated and vehicle-treated cells at the corresponding time point (* *p* ≤ 0.05; *** *p* ≤ 0.001; **** *p* ≤ 0.0001; ° *p* ≤ 0.05; °° *p* ≤ 0.01; °°°° *p* ≤ 0.0001).

Considering the complex scenario regarding the mechanisms involved in cell death induced by TBT in HTLV-1-infected cells offered by the results of our previous study [27], to obtain a comprehensive view of the occurring phenomena, cell death events induced in PB2/IL-2 and PB2/NO-IL-2 cells by the tributyltin compound were recapitulated. As a first approach to this issue, we decided to obtain information on the viability of PB2/IL-2 and PB2/NO-IL-2 infected cells soon after treatment. In fact, previous data on this aspect, collected at only 24 h post treatment using the Trypan Blue assay, showed a dramatic, dose-dependent decrease in cell viability both in PB2/IL-2 and PB2/NO-IL-2 cells, with a practically total disappearance of viable cells at 10 µM TBT. This suggested that at 24 h after treatment, most of the treated cells had already undergone a lytic death, although without the possibility to discriminate between a primary or a secondary form of lytic death. Thus, in this case, the classic Trypan Blue exclusion assay was also employed, but viability was assayed at 3, 6, and 12 h post treatment. Results, shown in Figure 2, clearly indicate that the total number of still intact/viable starts to decrease early after the contact with TBT both in PB2/IL-2 and PB2/NO-IL-2, but also that PB2/NO-IL-2 cells were much more sensitive to TBT compared to PB2/IL-2.

### 2.3. Time-Course Analysis of Cell-Death Induction by TBT in HTLV-1-Infected PB2/IL-2 and PB2/NO-IL-2 Cells via Real-Time Quantification of Fluorescent Cells Double-Stained with Green Caspase-3/7 and Red Cytotox Dyes

To go into further details about the kinetics of cell death induced by TBT in HTLV-1-infected cells, we then performed a time-course analysis of apoptotic and lytic cell death parameters, respectively, by means of the double staining of vehicle-treated and TBT-treated cells with green fluorescent caspase-3/7 dye and red fluorescent cytotox dye, followed by live-monitoring of the fluorescent cells in the cultures. For this purpose, PB2/IL-2 and PB2/NO-IL-2 cells, treated or not with TBT at the cytotoxic concentration of 10 µM, were subjected to a real-time automatic analysis of fluorescence during the ongoing course of the culture for a total time of 48 h in the stable environment of an Incucyte tissue culture incubator. Moreover, the results shown in Figure 2 indicate that, presumably, forms of lytic death that could not be ascribed to secondary necrosis occur early, especially in PB2/NO-IL-2, as a result of TBT treatment. Thus, also considering that a large amount of data show that HTLV-1-infected cells represent an inflammatory environment, to improve our knowledge on the type of cell death induced by TBT, some cell cultures in addition to TBT alone were cotreated with disulfiram, which is a well-known inhibitor of pyroptosis, a lytic form of regulated cell death (RCD). Data collected by the Incucyte high-throughput system were then analyzed using the dedicated software.

**Figure 3:**
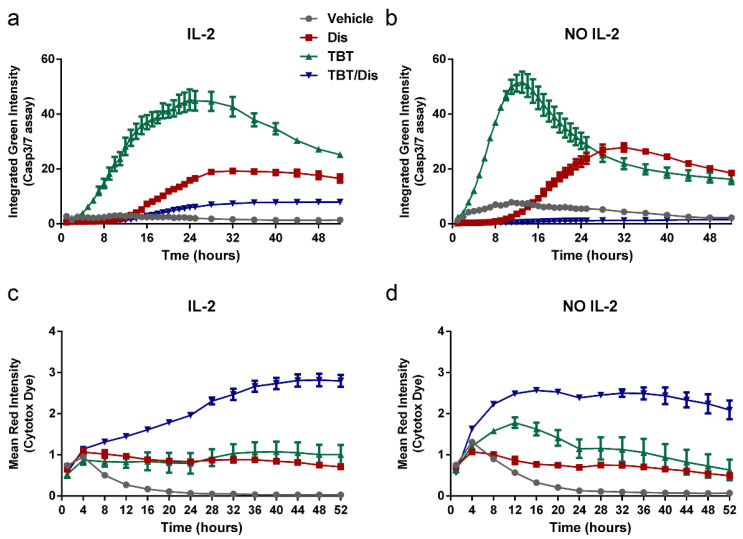
Real-time kinetic analysis of caspase-3/7 activation (**a**,**b**) and lytic cell death (**c**,**d**) in HTLV-1-infected PB2/IL-2 and PB2/NO-IL-2 cells treated with TBT and/or disulfiram. PB2/IL-2 (**a**,**c**) and PB2/NO-IL-2 (**b**,**d**) cells were treated with TBT 10 µM, disulfiram 20 µM (Dis), TBT plus disulfiram (TBT/Dis), or vehicle control and stained with CellEvent™ Caspase-3/7 Green Detection Reagent (**a**,**b**) or with Incucyte^®^ Cytotox Red Dye (**c**,**d**). Green fluorescence, associated with caspase-3/7 activation during apoptotic cell death (**a**,**b**), and red fluorescence, associated with loss of plasma membrane integrity during lytic cell death (**c**,**d**), were monitored in real time for 52 h using the IncuCyte^®^ SX1 live-cell analysis system. Fluorescent signals were quantified using the integrated IncuCyte^®^ analysis software (v2025A). Data are presented as mean ± SD from three independent experiments.

Impressive phenomena were observed via the simultaneous time-course analysis performed by real-time quantification of green caspase-3/7 dye fluorescent HTLV-1-infected PB2/IL-2 and PB2/NO-IL-2 cells subjected to different treatments. In this case, the curves of TBT-treated HTLV-1-infected cells show a remarkable increase in green fluorescent cells for both PB2/IL-2 and PB2/NO-IL-2 cells (Figure 3a,b), with a peak of fluorescence intensity much earlier and more marked for PB2/NO-IL-2 cells (Figure 3b), followed by a subsequent progressive decrease in green fluorescent cells, but still showing levels of green fluorescence higher than vehicle-treated cells. These kinetics seem to match with those of classic apoptotic cell death, and presumably with successive secondary necrosis occurrence. Moreover, intriguingly, it seems that the early, remarkable increase of caspase-3/7 green fluorescent PB2/NO-IL-2 TBT-treated cells overlaps with that of red fluorescent lytic TBT-treated cells detected simultaneously (Figure 3d). Nevertheless, unlike that observed for red fluorescent cells, no additional effects on green fluorescent cells were detected in disulfiram plus TBT PB2/IL-2 or PB2/NO-IL-2 treated cells, with fluorescence intensity comparable to those of vehicle-treated cells or even lower for PB2/NO-IL-2 cells (Figure 3a,b). In addition, disulfiram alone, in this case, induced remarkable levels of fluorescence intensity, but the peaks of the curves where delayed compared to those observed in TBT-treated caspase-3/7 fluorescent cells and coincident with the decreasing trend of fluorescence in the same cells, particularly for PB2/NO-IL-2 TBT-treated cells (Figure 3b).

The results, illustrated in the graphs of Figure 3, show overall decreasing trends of both green fluorescence and red fluorescence intensity, respectively, in vehicle-treated cells over the entire observation period in both PB2/IL-2 and PB2/NO-IL-2 cells, indicating negligible or absent levels of spontaneous cell death in HTLV-1-infected cells during the first 48 h in culture. Conversely, as shown in Figure 3c, in PB2/IL-2 TBT-treated infected cells, the curve of red fluorescent cells undergoing lytic cell death shows a quite stable trend in the first 24 h and a slightly increasing trend in the successive 24 h, with fluorescence intensity levels always higher in respect to vehicle-treated cells. For PB2/NO-IL-2 cells, on the other hand, the curve of lytic death shows an early increasing trend from the beginning of the observational time in culture in the first 12 h, with a slightly decreasing trend in the successive hours and, in any case, fluorescence intensity levels higher than those of control-treated cells. These trends are consistent with the occurrence of a type of primary lytic cell death early after the first contact of the HTLV-1-infected cell cultures with TBT, especially in PB2/NO-IL-2 cells, presumably followed by the occurrence of secondary necrosis starting from 24 h after treatment in cells that had previously undergone non-lytic forms of cell death, such as apoptotic RCD, as suggested by Figure 3b.

Unexpectedly, the kinetic curves of red fluorescence intensity levels for both PB2/IL-2 and PB2/NO-IL-2 cells treated with disulfiram alone were higher compared to those of vehicle-treated cells, with a trend overlapping that of TBT-treated cells for PB2/IL-2 cultures, while significantly lower levels of fluorescence intensity in comparison with those of TBT-treated cells were detected for PB2/NO-IL-2 cultures. Moreover, somewhat surprising were the additional effects of the combined—disulfiram plus TBT—treatment on red fluorescence intensity in HTLV-1-infected cells, with trends of the curves for both PB2/IL-2 and PB2/NO-IL-2 cell cultures resembling those observed in TBT-alone-treated cells, but constantly higher. These results indicate that disulfiram itself is endowed with a certain lytic death-inducing property toward HTLV-1-infected cells. The data obtained cannot clarify whether the observed phenomenon is related or not to the anti-pyroptotic activity of disulfiram; nevertheless, they clearly show that disulfiram does not inhibit the late form of lytic cell death induced by TBT.

### 2.4. Evaluation of the Effects of TBT Treatment on Apoptotic Response Parameters in HTLV-1-Infected Cell Lines: Assessment of Caspase-3 and Caspase-8 Activity Through Immunoblot Analysis

The time-course analysis of fluorescent cells double-stained with the cytotox red and caspase-3/7 green dyes designed a complex picture of cell death-related events in HTLV-1-infected cells, adding novel information to that given by previous results [27]. In any case, results of the live-monitoring of the fluorescent cells in the cultures agreed, at least in part, with those reported in our previous study [27], indicating the occurrence of a certain amount of apoptotic RCD in HTLV-1-infected cells in response to TBT treatment. To further confirm this hypothesis and provide additional details on possible mechanisms underlying the RCD induced by the tributyltin in HTLV-1-infected cells, activation of caspase-3 and caspase-8 following TBT treatment in PB2/IL-2 and PB2/NO-IL-2 cells by immunoblotting analysis was investigated.

**Figure 4:**
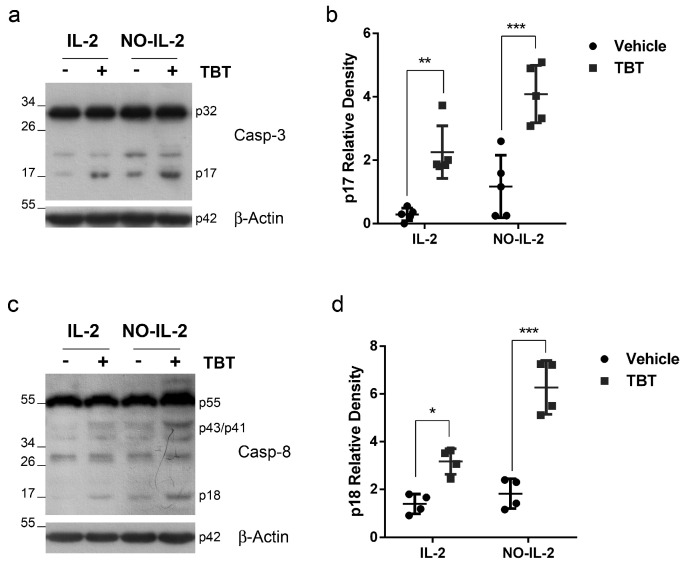
Western blot showing the effects of the TBT on the abundance of caspase-3 and caspase-8 related proteins in PB2/IL-2 and PB2/NO-IL-2 cells. Representative Western blot image showing the full length and cleaved forms of (**a**) caspase-3 (**c**) caspase-8 proteins in control (vehicle) and treatment (TBT) groups. Quantitative analysis of the cleaved forms p17 (**b**) and p18 (**d**) of caspase-3 and caspase-8, respectively, is also reported. Data are presented as the means ± SDs from at least three independent experiments, with asterisks indicating statistical differences: * *p* < 0.05, ** *p* < 0.01, *** *p* < 0.001.

A total of 5 × 10^6^ PB2/IL-2 and PB2/NO-IL-2 cells were treated for 2 h with 5 μM TBT. The results showed that in both PB2/IL2 and PB2/NO-IL2 treated cells, caspase-3 was clearly activated, as shown by the presence of the cleaved form p17, in comparison with the untreated cells (see Figure 4a, representative image). Moreover, the graph shown in Figure 4b, which summarizes the quantitative data derived from different experiments, demonstrates that this effect was much more evident in PB2/NO-IL-2 than in PB2/IL-2. Similar results were achieved when the activation of caspase-8 was assayed and the cleaved p18 form was identified and quantified (Figure 4c,d). Thus, these results were coherent with the occurrence of distinct levels of apoptosis in PB2/IL2 and PB2/NO-IL2 cells in response to TBT treatment.

### 2.5. Evaluation of the Effects of TBT Treatment on Pyroptotic Response Parameters in HTLV-1-Infected Cell Lines: Analysis of (i) Inflammatory Gene Expression, (ii) Cell Death Detected by Hoechst Staining Following Disulfiram Cotreatment, and (iii) Gasdermin-D Activation

The time-course analysis of HTLV-1-infected fluorescent cells double-stained with Red Cytotox and Green Caspase-3/7 Dyes and treated with TBT following pretreatment with or without disulfiram suggested that, in addition to apoptotic RCD or other forms of cell death, the pyroptotic form of RCD could also presumably be implicated in cell death induced by TBT in PB2/IL-2 and PB2/NO-IL-2 cells. To investigate whether HTLV-1-infected cells were actually sensitive to induction of this lytic form of cell death by TBT, different pyroptosis related parameters were assayed.

**Figure 5:**
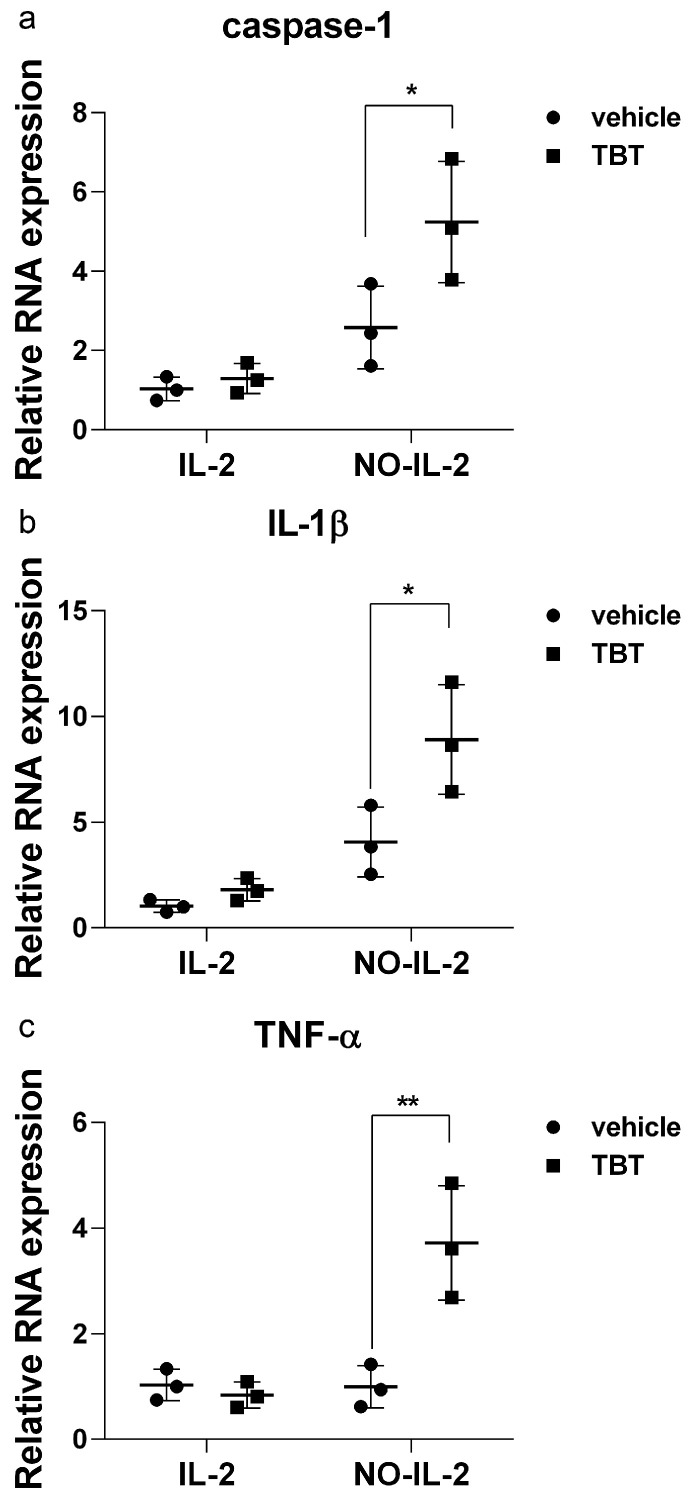
Relative mRNA expression levels of pyroptosis-related genes in HTLV-1-infected PB2/IL-2 and PB2/NO-IL-2 cells following TBT treatment. PB2/IL-2 and PB2/NO-IL-2 cells were treated with TBT (10 µM) or vehicle control, and total RNA was extracted and analyzed by RT-qPCR. Relative mRNA expression levels of (**a**) caspase-1, (**b**) IL-1β, and (**c**) TNF-α are shown. Expression levels were normalized to Actin beta RNA and calculated using the 2^−ΔΔCT^ method. Data represent mean ± SD of three independent experiments. Statistical significance between TBT-treated and vehicle-treated cells was evaluated by one-way ANOVA followed by Bonferroni’s multiple comparison test (* *p* < 0.05, ** *p* < 0.01).

Considering that pyroptosis is characterized as an inflammatory lytic cell death that can be triggered by caspase-1 in response to inflammosome activation, a comprehensive picture of a possible pro-pyroptotic environment in PB2/IL-2 and PB2/NO-IL-2 cells was first assayed by quantitative, comparative analysis of inflammatory gene expression in the two cell lines using RT-qPCR. To summarize this issue, mRNA levels of caspase-1, interleukin 1 beta (IL-1β), and tumor necrosis alpha (TNF-α), implicated in different roles in the pyroptosis pathway, were assayed. Results, shown in Figure 5, demonstrate that mRNA levels for caspase-1, the primary identified pyroptosis-triggering protein, were higher in both PB2/IL-2 and PB2/NO-IL-2 TBT-treated cells in comparison with vehicle-treated cells, showing for PB2/NO-IL-2 cells a higher, more significant increase in relative expression of this caspase in treated cells compared to control cells. Conversely, mRNA levels for IL-1β and TNF-α were significantly higher in TBT-treated cells than those of vehicle-treated cells only in PB2/NO-IL-2 cells and not in PB2/IL-2 cells. These results seem coherent with a lower pro-pyroptotic proneness in response to TBT for the PB2/IL-2 environment in comparison with the PB2/NO-IL-2 one.

To further investigate the possible involvement of pyroptosis in cell death induced by TBT in PB2/IL-2 and PB2/NO-IL-2 cells, a morphological analysis following Hoechst staining in cells treated for a short time with TBT and co-treated or not with disulfiram was then carried out. In fact, this microscopy technique allows the visualization of altered nuclear morphologies—consisting of nuclear condensation and fragmentation—that similarly occur in pyroptotic and apoptotic cells, and intense blue fluorescence in still intact dying cells due to membrane pore formation, typical of pyroptotic cells. Note that in this set of experiments, the short-time kinetic was chosen based on preliminary data indicating progressive cell debris accumulation in successive times.

**Figure 6:**
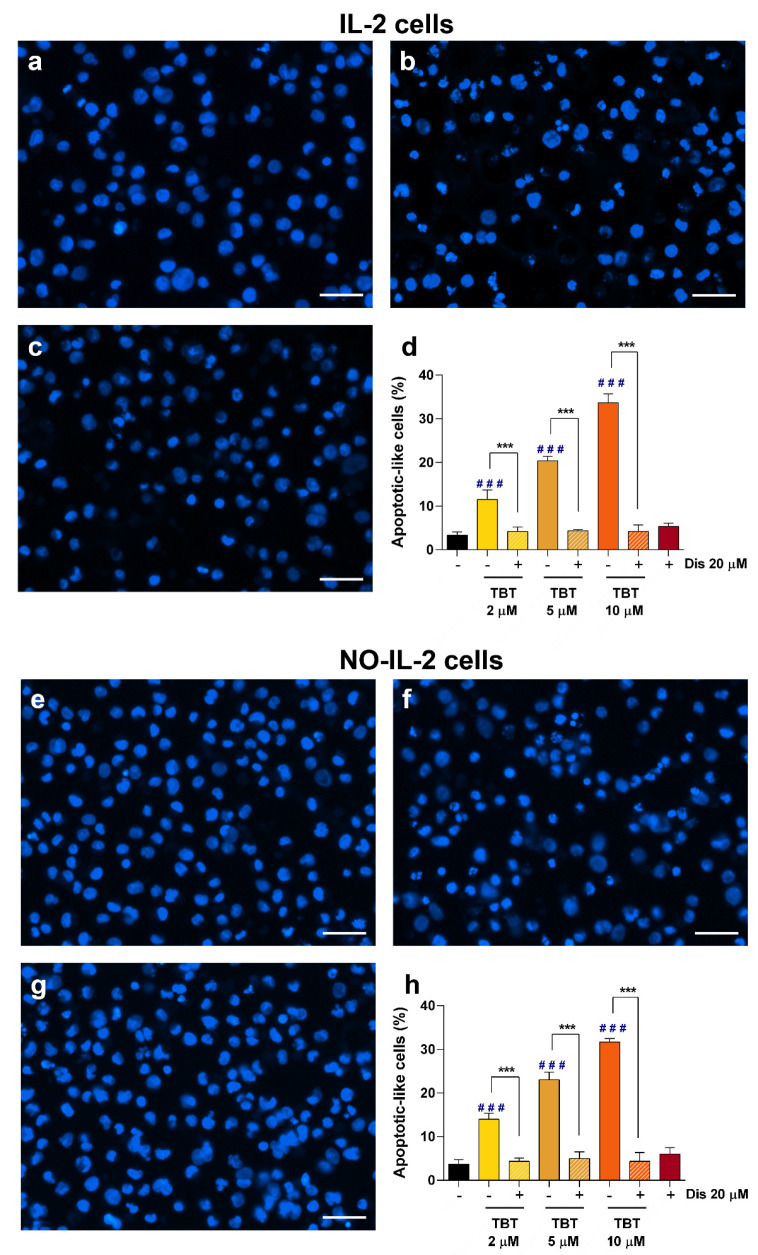
Effect of pre-treatment with 20 µM disulfiram for 1 h on the induction of cells with altered nuclei morphology by treatment with TBT for 6 h in PB2/IL-2 cells or for 3 h in PB2/NO-IL-2 cells, as detected by the Hoechst nuclei staining technique. Representative images show control PB2/IL-2 (**a**) or PB2/NO-IL-2 (**e**) cells, PB2/IL-2 (**b**) or PB2/NO-IL-2 (**f**) cells treated with 5 μM TBT, and PB2/IL-2 (**c**) or PB2/NO-IL-2 (**g**) cells pretreated with 20 μM disulfiram before 5 μM TBT. The graph summarizes the percentages of the PB2/IL-2 (**d**) or PB2/NO-IL-2 (**h**) cells showing altered nuclei after pre-treatment or not with disulfiram and then treatment with 2, 5, and 10 µM TBT. Data in the graph are expressed as mean values ± SD obtained from three independent experiments. Blue hashtags above the bars indicate statistically significant differences versus control (black bar, ### *p* < 0.001), whereas asterisks above connecting lines indicate statistically significant differences between cells treated with TBT alone and the corresponding groups pretreated with disulfiram (*** *p* < 0.001). Scale bars = 25 µm. Microscope objective: 20×.

The results shown in Figure 6c reveal a notable reduction in PB2/IL-2 cells with altered nuclear morphology following pre-treatment with the pyroptosis inhibitor disulfiram compared to cells treated with TBT alone. This phenomenon can be well appreciated by the numerical results represented in the graph, which show a clear dose-dependent induction of cells with nuclear morphology alteration, as a marker of early death, at all the concentrations of TBT assayed, and a corresponding highly significant inhibition exerted by pre-treatment with disulfiram on these effects (Figure 6d). When PB2/NO-IL-2 cells were subjected to the same protocol of pre-treatment with caspase inhibitors utilized for PB2/IL-2, the results showed that disulfiram was able to cause a well appreciable, highly significant reduction of cells showing nuclear morphology alteration following TBT treatment (Figure 6h). In this set of experiments performed both in PB2/IL-2 and PB2/NO-IL-2 cells, using disulfiram as pyroptosis inhibitor and a short-time kinetic of treatment, disulfiram alone did not modify the nuclear morphology of still-intact cells. Overall, results of experiments performed using the Hoechst staining technique for evaluating the nuclei morphology alteration confirm that presumably pyroptosis, at least partially, contributed to RCD caused by TBT in HTLV-1-infected cells.

**Figure 7:**
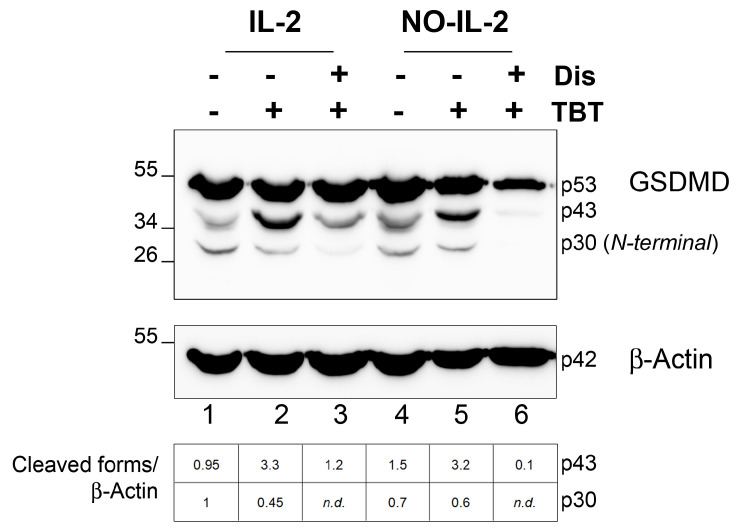
Analysis of gasdermin D (GSDMD) activation by Western blot in PB2/IL-2 and PB2/NO-IL-2 cells treated with TBT, with or without disulfiram pre-treatment. Representative Western blot showing the full-length form of GSDMD and the cleaved p43 and N-terminal p30 fragments in control cells, cells treated with TBT, and cells pre-treated with disulfiram before TBT exposure. β-Actin was used as a loading control. Numbers below the lanes indicate densitometric values of the cleaved p43 and p30 forms normalized to β-actin. n.d., not detectable.

Another pyroptosis-related parameter investigated in TBT-treated HTLV-1-infected cells was the activation of gasdermin D (GSDMD), notoriously involved as key executioner step for the consequent pore formation in the cell membrane by part of the generated N-terminal fragment of GSDMD. Activation of GSDMD was detected by immunoblot analysis of the full length and the cleaved p43 and p30 fragments of the protein by means of a Western Blot assay. Results obtained in a representative experiment of the three performed with identical results demonstrate, as shown in Figure 7, that basal levels of GSDMD activation were actually detected in both vehicle-treated HTLV-1-infected cell lines, and that, in any case, both in PB2/IL-2 and PB2/NO-IL-2 cells, this protein was overall activated in response to TBT treatment. In addition, disulfiram remarkably inhibited this activation. However, this overall activation was exclusively imputable to the formation of the p43 cleaved form of GSDMD, while levels of the p30 cleaved form were even lower than that detected in the vehicle-treated cells. Considering that the p30 cleaved form of GSDMD is the executer form of the protein that is responsible for the formation of the membrane pores, while the p43 cleaved form of the protein is that which negatively controls the execution of pyroptosis by hindering the action of p30, these results exclude the involvement of GSDMD in the induction of a pyroptotic, lytic-type death by TBT in HTLV-1-infected cells.

### 2.6. Effects of TBT on the Autophagic Response in HTLV-1-Infected Cells

**Figure 8:**
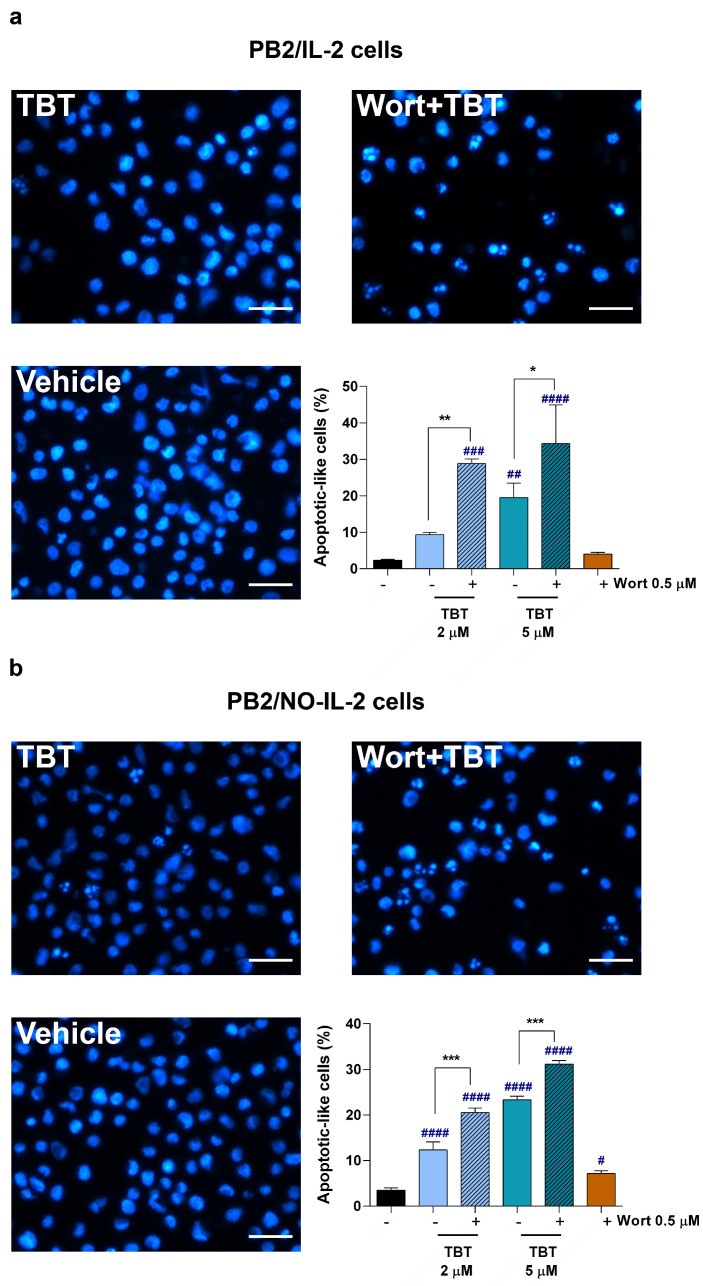
Effect of wortmannin pre-treatment on TBT-induced alterations of nuclear morphology detected by Hoechst nuclear staining in HTLV-1-infected PB2/IL-2 and PB2/NO-IL-2 cells. Representative images show vehicle-treated cells, cells treated with 5 μM TBT, and cells pre-treated with 0.5 μM wortmannin for 1 h 30 min before exposure to 5 μM TBT in PB2/IL-2 cells (**a**) and PB2/NO-IL-2 cells (**b**). PB2/IL-2 cells were treated with TBT for 6 h, whereas PB2/NO-IL-2 cells were treated for 3 h. Graphs summarize the percentages of cells showing apoptotic-like nuclear morphology after pre-treatment or not with wortmannin followed by exposure to 5 or 10 μM TBT. Data are expressed as mean values ± SD obtained from three independent experiments (*n* = 3) performed in triplicate. Blue hashtags indicate statistically significant comparisons versus vehicle-treated control cells, whereas asterisks under connection bars indicate statistically significant comparisons between corresponding TBT-treated groups in the absence or presence of wortmannin (* *p* ≤ 0.05, ** *p* ≤ 0.01, *** *p* ≤ 0.001; # *p* ≤ 0.05, ## *p* ≤ 0.01, ### *p* ≤ 0.001, #### *p* ≤ 0.0001). Scale bar = 25 μm. Objective magnification: 20×.

Based on the known mutual interactions between various types of RCD and autophagy, it was considered worthwhile to investigate whether an autophagic response was in some way involved in TBT-induced cell death. To this purpose, a total of 2.5 × 10^5^ PB2/IL-2 cells were pre-treated with wortmannin, an irreversible inhibitor of autophagy targeting class III phosphatidylinositol 3-kinase, for 1 h and 30 min, and successively treated for 6 h with 5 and 10 µM TBT. Microscopy analysis of nuclei morphology using the Hoescht staining method indicated that the combination of TBT 5 µM after a pre-treatment with wortmannin 0.5 µM caused an increase in the percentage of cells showing altered nuclear morphology compared to cells treated with TBT alone (Figure 8a). The results, summarized in the graph, show that cells pre-treated with wortmannin undergo a significant increase of cells with altered nuclei in respect to those treated with 5 and 10 µM TBT alone.

When PB2/NO-IL-2 cells underwent the same experimental procedures, the results, shown in the graph, confirm the clear enhancing effect exerted by wortmannin towards insurgence of nuclear morphology alteration induced by TBT also in this HTLV-1-infected cell line (Figure 8b). In addition, the representative images allow us to appreciate that the potentiation given by wortmannin of the effect caused by 5 µM TBT on nuclei morphology alteration was associated with an evident decrease in the number of intact cells both in PB2/IL-2 and PB2/NO-IL-2 cells. On the other side, the same experiments demonstrated that wortmannin alone was not toxic both for PB2/IL-2 and PB2/NO-IL-2 cells.

**Figure 9:**
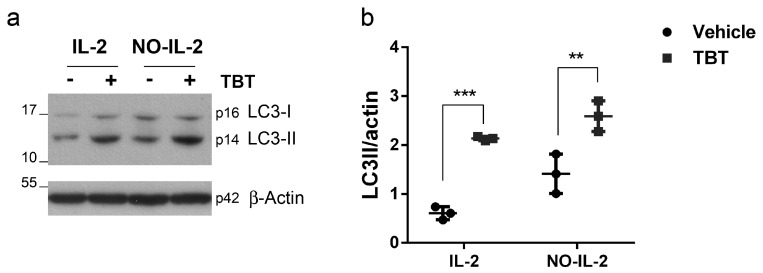
Western blot showing the effects of TBT on LC3 protein conversion in PB2/IL-2 and PB2/NO-IL-2 cells. Representative Western blot image showing LC3-I (p16) and LC3-II (p14) protein abundance in PB2/IL-2 and PB2/NO-IL-2 cells treated with vehicle (control) or TBT (**a**). β-Actin was used as loading control. Quantitative analysis of the LC3-II/β-actin ratio following Western blot analysis is shown in panel (**b**). Data are presented as mean ± SD from three independent experiments (*n* = 3). Statistical significance is indicated as follows: ** *p* < 0.01, *** *p* < 0.001.

These results suggested that presumably an autophagic response to counteract cell death induction by TBT was activated in HTLV-1-infected cells, and that inhibition of autophagy by wortmannin resulted in enhanced levels of cell death following TBT treatment. To corroborate this suggestion, autophagy was then directly assayed in response to TBT in HTLV-1-infected cells through immunoblot using antibodies specific for the autophagic protein LC3. Figure 9 shows the conversion of LC3-I p14 cytosolic to LC3-II p16 in autophagosome membrane. The loading control actin was equal in all the samples (Figure 9). The conversion of LC3-I p14 to LC3-II p16 in vehicle-treated PB2/NO-IL2 was higher in respect to those observed in vehicle-treated PB2/IL-2 cells. In PB2/IL-2 and PB2/NO-IL2 TBT treated cells the conversion to LC3-II p16 was higher in respect to those observed in vehicle-treated cells. These results clearly confirmed that an autophagic response was activated in HTLV-1-infected cells following TBT treatment.

## 3. Discussion

The results we obtained during this phase of our study allowed us to add further information on the effects of organotin compounds on HTLV-1-infected cells. First, we demonstrated that TBT, in addition to exerting a cytotoxic effect in HTLV-1-infected cells, was also able to significantly reduce viral gene expression early after cell contact. Such an early inhibitory effect suggests that decreased levels of viral RNA expression, especially for genes encoding Tax and HBZ HTLV-1 oncoproteins, was not simply reflecting a block of mRNA synthesis due to an advanced status of cell death. In addition, mRNA levels of viral genes are not expressed as absolute but as relative values, normalized to 18S. This should exclude the notion that downregulation of viral gene expressions induced by TBT in PB2/NO-IL-2 cells simply reflects a certain reduction in cells with an intact membrane at 6 h after treatment. Importantly, moreover, inhibition of viral genes expression by TBT occurred in a cell line-specific manner. The major and complex role of Tax and HBZ in ATL progression and in the perpetuation of leukemia in ATL patients, as well as, consequently, their pivotal role as therapeutic targets for potential anti-ATL chemotherapic agents, has been documented in several studies, as reviewed in El Hajj [14]. Regarding our results, it is worth noting that the basal mRNA level for Tax and HBZ viral genes showed different entities in PB2/IL-2 and PB2/NO-IL-2 cell lines, being more relevant in the latter. This is in accordance with a more advanced state of pre-malignancy along the HTLV-1-driven leukemogenesis pathway, thus strengthening the credibility of the cellular model of ATL transformation we generated by selecting cell lines after different times from in vitro HTLV-1 infection and at different states of IL-2 dependence/independence. In fact, inhibition of Tax and HBZ gene expression by TBT treatment was clear when the basal levels of gene expression for the same proteins were higher, i.e., in PB2/NO-IL-2 cells, while practically undetectable in the counterpart cells, resembling the preceding state of leukemogenesis consisting in HTLV-1-driven immortalization. A translational reading of these results suggests that a possible optimal time gap for an organotin-compound-based anti-ATL treatment occurs as soon as symptoms of HTLV-1-driven leukemogenesis appear and not before or much later. Such a time gap could be useful to benefit from both a direct cytotoxic effect of the compound and an indirect anti-viral-leukemogenic effect, determined by the depletion of the expression of viral genes mainly involved in the oncogenesis activity exerted by HTLV-1 in a synergistic way.

A second issue on which this study is focused is related to the efforts to identify the specific characteristics of cell death induced by TBT in HTLV-1-infected cells, and to try to place them within the frame of a known type of cell death. An important contribution to this issue was provided by the experiments performed using the Incucyte^®^ live-cell analysis system for real-time evaluation, which undoubtedly clarified the kinetics and the amounts of events occurring in the cells in response to exposure to TBT in the different experimental conditions, as assayed by means of this technique, i.e., caspase 3/7 activation and membrane pore formation.

Regarding apoptotic RCD, the kinetics of occurrence and the quantification of cells showing caspase-3/7 activation, as reported by the live-cell analysis, together with results of immunoblot analysis for caspase-3 and caspase-8 activity, confirmed that this type of cell death is implicated in cytotoxicity of TBT towards HTLV-1-infected cells, with a higher incidence in PB2/NO-IL-2 cells than in PB2/IL-2, as also suggested by our previous study [27]. In addition, results of kinetics of membrane pore formation, undoubtedly demonstrated that also a lytic form of cell death insurge early after TBT treatment in both PB2/IL-2 and PB2/NO-IL-2 cells, i.e., at times which exclude a possible occurrence of secondary necrosis in cells which previously underwent to apoptosis. Again, occurrence of lytic cell death was more pronounced and faster in PB2/NO-IL-2 cells compared to PB2/IL-2 cells after TBT treatment, confirming that the former cell line is more sensitive to cell death induction by TBT, as also shown in experiments using the Trypan Blue exclusion test in the framework of this study and in results obtained in our previous study. Detection of still-intact Cytotox-stained cells persisted over time in both cell lines after treatment, showing a higher decline after a higher initial boost in PB2/NO-IL-2 cells and indicating that new cells were subjected to lytic death even after 20/24 h and later after treatment. However, at these times, it cannot be excluded that cells undergoing lysis are cells subjected to secondary necrosis. Considering that membrane pore formation is a typical event occurring in pyroptotic cell death, in addition to vehicle-treated cells and TBT-treated cells, our live-cell experiments with Cytotox staining were extended also to cells treated with disulfiram alone—as a pyroptosis inhibitor—and disulfiram in addition to TBT. These experimental conditions, however, did not allow us to confirm or exclude that TBT induces pyroptosis in HTLV-1-infected cells. In fact, we found that disulfiram is able to induce on its own a lytic form of cell death in HTLV-1-infected cells, and that it can produce an additional and not inhibitory effect with TBT towards lytic death in the same cells. Thus, the issue of whether pyroptosis occurs in response to TBT in HTLV-1-infected cells was addressed with different experimental approaches. A first experimental approach to this issue was the direct ascertainment of whether the cellular environment we generated and used for performing our experiments on the occurrence of pyroptosis in response to TBT, i.e., PB2/IL-2 and PB2/NO-IL-2 cells, were compatible with a pyroptotic/inflammatory environment. Analysis of inflammatory cytokines following TBT treatment gave an affirmative response, showing that PB2/NO-IL-2 cells, i.e., the cell line that was more prone to lytic death in response to TBT, were also more prone to the production of pro-pyroptotic cytokines in response to TBT. Then, another experimental approach to the same issue was the repetition of experiments using disulfiram as a pyroptosis inhibitor but employing a different methodological tool to detect cell death, such as microscopy analysis following Hoechst staining. In this case, the alteration of nuclear morphology in response to TBT was actually inhibited by disulfiram in both PB2/IL-2 and PB2/NO-IL-2 cells, suggesting that the pyroptotic pathway should also be implicated in the induction of cell death by TBT in HTLV-1-infected cells. When, however, another technical approach, such as the activation of GSDMD as the principal executer event for pyroptotic pore formation, was utilized to ascertain the involvement of pyroptosis in cell death induced by TBT in PB2/IL-2 and PB2/NO-IL-2 cells, unfortunately we could not provide this element as an additional proof of occurrence of pyroptosis in TBT-treated HTLV-1-infected cells. We do not have a clear interpretation for this apparent inconsistency; however, we must take into consideration the possibility that, in our case—i.e., with HTLV-1-infected cells and an organotin compound—for unknown reasons the pyroptosis induction does not utilize the classical pathway of GSDMD activation, but rather a non-classical pathway. Interestingly, it has been found that another gasdermin, i.e., gasderimin-E, can be activated by caspase-3, thus connecting the apoptotic and the pyroptotic pathways in cancer cells [28]. Alternatively, the lytic death induced by TBT in HTLV-1-infected cells is similar to pyroptosis, but it is not pyroptosys itself [29]. Further studies are necessary to settle this issue. As for experiments on pyroptosis, it is notable that results we found using disulfiram present also a new scenario worthy of further investigation, consisting in the ascertainment of whether disulfiram itself, alone or in combination with other compounds, could be considered as a useful repurposed drug for the treatment of HTLV-1-driven diseases. This novel avenue, opened by the unexpected results of our study, is compatible with findings indicating that disulfiram forms complexes with metal ions and can exert anticancer action by inhibiting proteasome activity and targeting aldehyde dehydrogenase [30].

Finally, another aspect of this study involves the relationships among cell death and the autophagic response in TBT-treated HTLV-1-infected cell lines. The intricate interconnection between apoptosis and autophagy and its implications in tumorigenesis has been pointed out for years [31]. Induction of autophagy in tumor cells in response to antineoplastic agents, such as cisplatin, and the complex interplay between autophagy and apoptotic cell death in response to chemotherapy, have been reported already by several authors [29,32,33]. Moreover, it has also been demonstrated that the oncogenic Tax viral protein promotes the autophagy process in HTLV-1-infected cells [34]. Interestingly, findings that PB2/NO-IL-2 cells have basal levels of both Tax expression and LC3-I p14 to LC3-II p16 conversion compared to PB2/IL-2 cells are in line, once again, with our assumption that our HTLV-1-infected cell lines reproduce successive phases of HTLV-1 driven oncogenesis. Results reported in this study show that TBT can activate an autophagic response in HTLV-1-infected cells and that a pretreatment with the autophagy inhibitor wortmannin can increase levels of cells showing altered nuclei morphology following treatment with TBT. Thus, these results indicate that the intricate interplay between autophagy and apoptosis affects also the effects of TBT on cell death-related events in HTLV-1-infected cells; this organotin compound therefore exerts a double-faced function consisting of triggering at the same time pro-apoptotic signaling and anti-apoptotic, autophagy-dependent activity in these cells. Notably, these data lead also to considering autophagy inhibitors as promising compounds for a targeted combination treatment with organotin compounds against HTLV-1-infected cells. Indeed, the capacity of wortmannin to potentiate the cytotoxicity of TBT had already been shown in a previous study [10].

**Figure 10:**
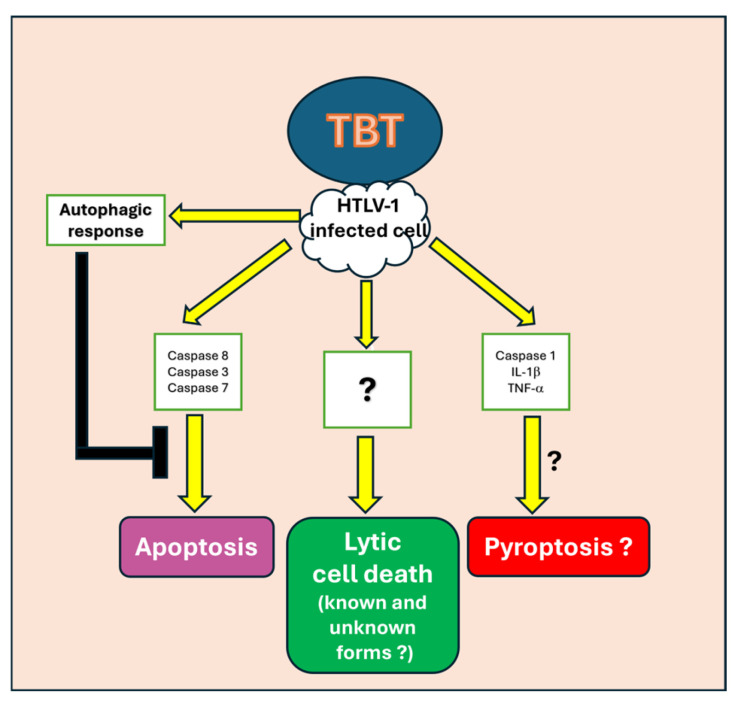
Schematic model of mechanisms of TBT-induced cell death in HTLV-1-infected cells. Where no question mark appears next to words/schemes of the model it means that these mechanisms have been experimentally validated during this study, while where a question mark appears next to words/schemes these mechanisms remain speculative.

A major limit of this study is that, although a large amount of data of death-related events on the effects of the TBT organotin compound towards HTLV-1-infected cells have been collected, it is not possible to conclusively classify death induced by TBT in these cells within a unique framework of regulated or unregulated form of cell death. Rather, it seems that a variety of known forms coexist. Notoriously, it is well recognized nowadays that a plethora of different forms of RCD exist, and another limit of the study is that not all the forms of RCD have been specifically examined. However, all those exhibiting characteristics more compatible with actors involved in the study, i.e., HTLV-1-infected leukemic-like cells and the TBT organotin compound, have been investigated; on the other hand, more than this was not possible in the course of a single study. Moreover, our results cannot also exclude that we are faced, among the other metal-specific forms of death, with a new tin-peculiar form of cell death (stannumptosis?). A schematic model summarizing the proposed mechanisms of TBT-induced cell death in HTLV-1-infected cells is reported in Figure 10. Future research is necessary to clarify all this and to verify the relevance of organotin compounds, alone or most likely in combination, in the treatment of diseases caused by HTLV-1.

## 4. Materials and Methods

### 4.1. Cells

PB2/IL-2 and PB2/NO-IL-2 HTLV-1-infected cell lines were generated and maintained as previously described [27]. Briefly, PB2/IL-2 cells were established following in vitro HTLV-1 infection of peripheral blood mononuclear cells and maintained in continuous culture in RPMI complete medium supplemented with IL-2. PB2/NO-IL-2 cells were subsequently derived by progressive selection toward IL-2 independence and stabilized in continuous culture in the absence of IL-2.

For experimental procedures, PB2/IL-2 and PB2/NO-IL-2 cells were thawed and cultured in suspension at a density of 4.5 × 10^5^ cells/mL in RPMI 1640 complete medium containing L-glutamine, 25 mM HEPES, penicillin (50 U/mL), streptomycin (50 U/mL), and 10% fetal bovine serum (FBS), in the presence or absence of 20 U/mL interleukin-2 (IL-2), respectively, as previously reported. All these reagents were obtained from Sigma-Aldrich (Saint Louis, MO, USA). Cells were maintained at 37 °C in a humidified atmosphere containing 5% CO_2_.

### 4.2. Chemicals, Inhibitors, and Antibodies

Tributyltin 2,2,2-trifluoroacetate, herein referred to as TBT, was synthesized at the Department of Chemistry “Ugo Schiff”, University of Florence, as previously described [10]. A 1 M stock solution was prepared in dimethyl sulfoxide (DMSO) and stored at −20 °C until use. Vehicle-treated control samples received corresponding amounts of DMSO.

Wortmannin (Selleckchem, Houston, TX, USA), a phosphoinositide 3-kinase inhibitor utilized to inhibit autophagic responses, was dissolved in DMSO at 100 mM and stored at −20 °C. Cells were pre-treated with 0.5 μM wortmannin for 1 h and 30 min before TBT exposure.

Disulfiram (Sigma-Aldrich, Saint Louis, MO, USA), employed as an inhibitor of gasdermin D pore formation, was dissolved in DMSO at 100 mM and stored at −20 °C. Cells were pre-treated with 20 μM disulfiram for 1 h before TBT exposure. This concentration was selected based on the previous literature [35] and preliminary experiments showing not-significant levels of cells with altered nuclear morphology up to this dose in our cells (see also Figure 6).

The following antibodies were employed for Western blot analysis: anti-cleaved caspase-3 (#9661, Cell Signaling Technology, Danvers, MA, USA), anti-caspase-3 (#19677-1AP, Proteintech, Chicago, IL, USA), anti-caspase-8 (#AF1650, R&D Systems, Minneapolis, MN, USA), anti-LC3 (#2775S, Cell Signaling Technology), anti-GSDMD (#97558, Cell Signaling Technology), and anti-β-actin (#66009-1, Proteintech). Horseradish peroxidase-conjugated secondary antibodies were obtained from Proteintech.

### 4.3. Cell Viability and Nuclear Morphology Analysis

Cell viability was assessed by Trypan Blue exclusion assay. Cell suspensions were mixed 1:1 with 0.4% Trypan Blue solution (Sigma-Aldrich) and viable/non-viable cells were manually counted using a Bürker chamber under phase-contrast microscopy (EVOS XL Imaging System, Thermo Fisher Scientifc, Waltham, MA, USA).

Nuclear morphology alterations were evaluated by Hoechst 33342 staining as previously described, with minor modifications. Cells (2.5 × 10^5^) were seeded in 24-well plates and exposed to treatments for 3 or 6 h according to experimental conditions. Cells were then collected, fixed with 4% paraformaldehyde, stained with Hoechst 33342 (Invitrogen, Carlsbad, CA, USA) at a final concentration of 10 μg/mL, and analyzed by fluorescence microscopy (Nikon Eclipse TE2000-S, Kingston upon Thames, UK) using a 20× objective. Chromatin condensation, nuclear fragmentation, and apoptotic body formation were considered apoptotic-like nuclear morphology parameters.

### 4.4. RT-qPCR Analysis

Total RNA extraction, reverse transcription, and RT-qPCR procedures were performed as previously described [36], with minor modifications. Briefly, total RNA was extracted using TRIzol reagent (Invitrogen), and cDNA synthesis was carried out using Maxima First Strand cDNA Synthesis Kit for RT-qPCR (Thermo Scientific, Waltham, MA, USA). Quantitative PCR amplification was performed using Maxima SYBR Green Master Mix (Thermo Scientific) on a CFX96 Real-Time PCR Detection System (Bio-Rad, Hercules, CA, USA). Expression levels of viral genes (Tax, Pol, HBZ) and host genes related to inflammatory and pyroptotic responses (IL-1β, TNF-α, CASP1) were normalized to 18S or Actin beta (ACTB) expression, respectively, and calculated using the 2^−ΔΔCt^ method, with the lowest expressed sample as the calibrator. Primers were purchased from Eurofins (Luxembourg, Nantes, Bruxelles) and sequences are reported in Table 1.

### 4.5. Western Blot Analysis

Western blot analysis was performed as previously described [37], with minor modifications. Briefly, after treatment, 5 × 10^6^ cells/sample were collected and lysed in hypotonic buffer containing protease inhibitors. Protein concentration was determined by DC Protein Assay (Bio-Rad). Equal amounts of proteins (30–40 μg/sample) were separated by SDS-PAGE and transferred onto nitrocellulose membranes. Following blocking and incubation with primary antibodies overnight at 4 °C, membranes were incubated with the corresponding horseradish peroxidase-conjugated secondary antibodies. Protein detection was performed using LiteAblot Extend chemiluminescent substrate (EuroClone, Pero, Italy). Chemiluminescent signals were acquired either by autoradiographic film exposure or digitally using the ChemiDoc Imaging System (Bio-Rad, Hercules, CA, USA), depending on the experimental setting. Densitometric analyses were performed using ImageJ software (v1.54) and protein abundance was normalized to β-actin.

### 4.6. Real-Time Analysis of Cell Death by IncuCyte^®^ Live-Cell Imaging

Real-time monitoring of cell death kinetics was performed using an IncuCyte^®^ SX1 Live-Cell Analysis System (Sartorius, Ann Arbor, MI, USA). PB2/IL-2 and PB2/NO-IL-2 cells were seeded in 96-well plates and exposed to experimental treatments. For each cell line, four experimental conditions were analyzed: vehicle-treated cells, TBT-treated cells, disulfiram-treated cells, and cells pre-treated with disulfiram followed by TBT exposure. To simultaneously monitor apoptotic and lytic cell death events, CellEvent™ Caspase-3/7 Green Detection Reagent (Invitrogen™, Thermo Fisher Scientific, Waltham, MA, USA) and IncuCyte^®^ Cytotox Red Dye (Sartorius, Ann Arbor, MI, USA) were directly added to culture medium according to the manufacturers’ instructions at final concentrations of 6 μM and 1.5 μM, respectively [27]. Plates were maintained within the IncuCyte^®^ instrument housed in a humidified incubator at 37 °C with 5% CO_2_. Phase-contrast and fluorescence images were automatically acquired every 4 h for up to 52 h using a 10× objective. Fluorescence exposure times were set at 400 ms for the green channel and 300 ms for the red channel. Image analysis parameters were optimized according to the manufacturer workflow and quantitative analyses were performed using integrated IncuCyte^®^ image analysis software.

### 4.7. Statistical Analysis

Statistical analyses were performed using GraphPad Prism version 9.0 software (GraphPad Software, San Diego, CA, USA). Data are presented as mean ± standard deviation (SD) obtained from at least three independent experiments. Statistical significance was evaluated by one-way ANOVA followed by Bonferroni post hoc correction, as appropriate. Statistical comparisons and significance thresholds are indicated in corresponding figure legend.

## 5. Conclusions

Overall, data collected during the experiments reported in this study outline typical features of the complex profile of cell death induced by TBT in HTLV-1-infected cells that could be useful for future studies aimed at fully defining this profile and verifying the real potential of tin-based compounds to counteract diseases caused by HTLV-1.

## Figures and Tables

**Table 1:** List of primer sequences.

Target Gene	GenBank Number	Primer Sequence (5′ → 3′)	
*Tax*	NC_001436.1	Sense Antisense	CACCCTTTTCCAGCCTGCTA GGAGTGGTGAGGGTTGAGTG
*Pol*	NC_001436.1	Sense Antisense	CAAAACCCAGCAAACCCCTG CCCACTGAATCTCGCCAAGT
*HBZ*	KF053885.1	Sense Antisense	GGAGACAAGGGGCTGAGAAG CCTCGCCTTCCAACTGTCTA
*18S*	NR_003286.2	Sense Antisense	GTAACCCGTTGAACCCCATT CCATCCAATCGGTAGTAGCG
*IL-1 β*	NM_000576	Sense Antisense	TTAAAGCCCGCCTGACAGA GCGAATGACAGAGGGTTTCTTAG
*CASP1*	NM_033292.4	Sense Antisense	TTTCCGCAAGGTTCGATTTTCA GGCATCTGCGCTCTACCATC
*TNF-α*	NM_000594	Sense Antisense	GCAGGTCTACTTTGGGATCATTG GCGTTTGGGAAGGTTGGA
*ACTB*	NM_001101.5	Sense Antisense	CCTCGCCTTTGCCGATCC ATCATCATCCATGGTGAGCTGG

## Data Availability

All data are contained within the article.

## Abbreviations

The following abbreviations are used in this manuscript:

HTLV-1	Human T-Lymphotropic virus type 1
WHO	World Health Organization
HNSCC	Head and neck squamous cell carcinoma
TBT	Tributyltin 2,2,2-trifluoroacetate
ATLL	Adult T-cell leukaemia/lymphoma
HAM/TSP	HTLV-1-associated myelopathy or tropical spastic paraparesis
PB2/IL-2	Stable IL-2 dependent cell line
PB2/NO-IL-2	Stable IL-2 independent cell line
RCD	Regulated cell death
IL-1β	Interleukin 1 beta
GSDMD	Gasdermin D
DMSO	Dimethyl sulfoxide
ACTB	Actin beta
